# *Helicobacter pylori* in Turkish Children with Dyspepsia: Diagnosis, Prevalence, Genotyping and Antibiotic Resistance

**DOI:** 10.1007/s12098-025-05635-2

**Published:** 2025-07-05

**Authors:** Derya Altay, Emre Karakaya, Duran Arslan, Kemal Deniz, Özgür Güran, Seçil Abay, Fuat Aydın

**Affiliations:** 1 Department of Pediatric Gastroenterology, Erciyes University, Faculty of Medicine, Kayseri, Turkey; 2 Department of Microbiology, Erciyes University, Faculty of Veterinary Medicine, Kayseri, Turkey; 3 Department of Pathology, Erciyes University, Faculty of Medicine, Kayseri, Turkey; 4 Department of Veterinary Microbiology, Erciyes University, Institute of Health Sciences, Kayseri, Turkey

**Keywords:** Antibacterial resistance, Dyspepsia, Genotyping, *Helicobacter pylori*, Histopathology

## Abstract

**Objectives:**

To investigate the clinical, culture-based, histopathological and molecular aspects, genotyping and antibiotic susceptibility patterns of *Helicobacter pylori* (*H. pylori*) infection in dyspeptic pediatric patients.

**Methods:**

Biopsy samples from 245 dyspeptic pediatric patients were used, with four samples taken from each patient. Two samples were used for bacteriological/molecular analysis, while the others were used for histopathological examination. The presence of the virulence genes babA2, cagA, cagE, iceA and vacA in *H. pylori* isolates was analyzed by PCR. The antibiotic susceptibility of the isolates to amoxicillin, clarithromycin, levofloxacin, metronidazole, rifampicin and tetracycline was analyzed using the gradient method (E-test). Histopathologically, the presence of *H. pylori* was detected by immunohistochemical staining with anti-*H. pylori* antibodies.

**Results:**

Bacteriological analysis revealed that 70 (28.57%) of 245 samples were positive for *H. pylori*, with 71 *H. pylori* isolates obtained from 70 samples (two isolates from one sample). Molecular analysis showed 81 (33.06%) samples to be positive, while histopathological examination revealed that 76 (31.02%) were positive for *H. pylori*. Immunohistochemical staining showed that 64 (79%) of the 81 PCR-positive samples were positive. Antibiotic susceptibility testing showed resistance to metronidazole in 55 (77.46%) of 71 isolates, 19 (26.76%) to clarithromycin, 10 (14.08%) to levofloxacin and 4 (5.63%) to rifampicin. All isolates were sensitive to amoxicillin and tetracycline.

**Conclusions:**

The data obtained are of great epidemiological importance. In addition, the results of antibiotic susceptibility test may help to select appropriate antibiotics for the treatment of the disease.

**Supplementary Information:**

The online version contains supplementary material available at 10.1007/s12098-025-05635-2.

## Introduction

*Helicobacter pylori*, the type species of the genus Helicobacter, is a Gram-negative, motile, spiral/curved and microaerophilic bacterium [1]. In 1984, Robert Warren and Barry Marshall first demonstrated the relationship between *H. pylori* and peptic ulcers [2]. Since then, *H. pylori* has been recognized as a major cause of peptic ulcers and a significant risk factor for gastric cancer [3]. The bacterium primarily colonizes the human gastric mucosa, often in the antrum, and it is estimated that around half of the world’s population is infected with this bacterium. The prevalence is particularly high in developing countries, with infection typically acquired in childhood, often in the family environment [4].

Invasive methods include bacterial isolation from gastric biopsy material obtained by endoscopy, histopathological examination and rapid urease testing. While culture-based examination focuses on bacterial isolation, histopathological analysis involves staining the biopsy material and assessing the tissue for the presence of *H. pylori*, inflammatory activity, glandular atrophy and intestinal metaplasia [4, 5].

As far as authors know, no study on culture-based testing, antibiotic susceptibility and genotyping of *H. pylori* in pediatric dyspepsia patients has been conducted in their region. Given this information, the current study investigated *H. pylori* infection in dyspeptic pediatric patients by clinical, culture-based, histopathological and molecular analysis as well as genotyping and antibiotic susceptibility of the *H. pylori* isolates found.

## Material and Methods

The study protocol was approved by the Clinical Research Ethics Committee of Erciyes University (2019/280, 17 April 2019) and was supported by the Scientific Research Project Group of Erciyes University (TSA-2020-10085).

Informed consent was obtained from the patients’ parents. Upper gastrointestinal endoscopy (using a Fujinon 4400-HD-EG530FP model endoscopy system) was performed on pediatric patients presenting with dyspeptic symptoms to the Erciyes University Faculty of Medicine, Department of Pediatric Gastroenterology. Male and female patients aged 6–18 y with untreated dyspeptic complaints who underwent upper gastrointestinal endoscopy and biopsies were taken from the antrum of the stomach were included in the study, while patients with endoscopy indications other than gastritis were excluded from the study. During endoscopy, four biopsy samples were taken from the antrum, two for histopathological examination by the pathology department and two for bacteriological and molecular analysis, which were stored at -80 °C in the Department of Microbiology of the Faculty of Veterinary Medicine of Erciyes University.

Biopsy samples stored in Brain Heart Infusion Broth (CM1135, Thermo Fisher Scientific, USA) were homogenized using a sterile glass pestle. After incubation, colonies suspected to be *H. pylori* were analyzed for phenotypic tests (Gram stain, morphology, motility, catalase, oxidase and urease activities) [6].

DNA was isolated from both the *H. pylori* isolates (from culture-based and from the gastric biopsy samples) using the EasyPure^®^ Genomic DNA Kit (EE101-12, TransGen, China) according to the manufacturer’s instructions.

Species-specific PCR amplifying the phosphoglucosamine mutase gene (glmM) was used to confirm *H. pylori* isolates identified by phenotypic testing and to detect *H. pylori* in gastric biopsy samples [7]. PCR products were analyzed by 1.5% agarose gel electrophoresis (Biomax, Agarose, lot number 124543PR, Prona, European Economic Community) and visualized under a UV transilluminator (GChemi XRQ; Syngene, Cambridge, UK). Bands of 294 bp were considered positive for *H. pylori*.

Each of the ten virulence genes of *H. pylori* was analyzed with a single PCR. The amplified products were electrophoresed on a 1.5% agarose gel, and product sizes of 271 bp, 349 bp, 508 bp, 247 bp, 229 bp, 567/642 bp, 259/286 bp, 190 bp, 187 bp and 213 bp were considered positive for babA2, cagA, cagE, iceA1, iceA2, vacA m1/m2, s1/s2, s1a, s1b and s1c, respectively [8–10].

Genotyping was performed using the Enterobacterial Repetitive Intergenic Consensus-Polymerase Chain Reaction (ERIC-PCR), as described by Versalovic et al. [11]. Primers 1R and 2 were used for PCR. Electrophoresis of the amplified products was performed on a 2% agarose gel. The banding patterns of ERIC-PCR were analyzed using BioNumerics software version 7.6 (AppliedMaths, Ghent, Belgium). The similarities between strains were calculated using the Dice coefficient and a dendrogram was generated using the Unweighted Pair Group Method with the Arithmetic Average (UPGMA) clustering algorithm. A similarity index of > 80% was used to define clusters [12].

The gradient method was used to determine the antibiotic susceptibility of 71 *H. pylori* isolates from biopsy samples. The antibiotics tested included amoxicillin (AC), clarithromycin (CH), levofloxacin (LE), metronidazole (MZ), rifampicin (RI) and tetracycline (TC) (Biomerieux, France). The minimum inhibitory concentration (MIC) values were interpreted according to the limits given in the EUCAST guidelines [13]. The MIC values (mg/L) of amoxicillin, clarithromycin, tetracycline, metronidazole, levofloxacin, and rifampicin were ≤ 0.125, ≤ 0.5, ≤ 1, ≤8, ≤ 1, and ≤ 1, respectively.

The *Helicobacter pylori* ATCC 700824 strain was used as the reference strain for the culture-based examination and molecular analysis.

The gastric biopsies were fixed with 10% formalin and tissue processing was performed using the Leica ASP300 tissue processor according to the standard protocol for tissue processing in authors’ pathology department. From the paraffin-embedded blocks, 5 μm thick tissue sections were cut with a microtome and mounted on standard slides for hematoxylin-eosin staining and on polylysine L-coated slides for immunohistochemical staining. Immunohistochemical staining was performed on the Ventana Benchmark Autostainer with the polyclonal antibody against rabbit *H. pylori* (Zeta Corporation, Arcadia, CA, USA) at a dilution of 1/200.

Gastric biopsies stained with hematoxylin-eosin were examined under a light microscope using the updated Sydney system for the presence of *H. pylori*, chronic and active inflammation, intestinal metaplasia and atrophy. Immunostaining was performed by a single pathologist for the presence of *H. pylori*. Brown staining in the sections was considered positive, indicating the presence of *H. pylori*. The pathologist was blinded to the PCR or culture results.

Statistical analysis was performed using IBM SPSS v.26. Descriptive statistics for numeric variables were reported as mean and standard deviation or median and interquartile range. Categorical variables were presented as frequencies and percentages. The chi-square test was used to assess the relationships or differences between categorical variables. The normal distribution of the data was assessed using hypothesis tests and graphical methods (Shapiro-Wilk test, histograms, QQ plots, etc.). For comparisons between two groups, a t-test for independent samples was used if the assumptions of normality were met; otherwise, the Mann-Whitney U test was used. A significance level of *p* < 0.05 was considered statistically significant.

## Results

A total of 245 pediatric patients (160 females, mean age 14 *±* 3.1 y) were included in the study. The most common presenting symptom was abdominal pain (172 patients, 70%), followed by vomiting (57 patients, 23%) and dysphagia (15 patients, 6%).

Among the endoscopic findings, hyperemia of the stomach with accompanying antral nodule formation was most frequently observed, occurring in 46 patients (56.8%). When comparing the endoscopic findings between the PCR-positive and PCR-negative groups, gastric hyperemia was the most common finding in both groups (63/81, 77.8% and 108/164, 65.9%, respectively).

In the molecular analysis (PCR) performed on DNA extracted from the biopsy samples, 81 (33%) of the 245 samples were found to be positive for *H. pylori*. In addition, PCR confirmed that all 71 isolates from 70 biopsy samples previously identified as *H. pylori* by phenotypic testing (including 2 *H. pylori* isolates from 1 sample) were *H. pylori*.

Histopathological examination revealed *H. pylori* positivity in 76 (31%) patients. *H. pylori* positivity was confirmed by hematoxylin and eosin staining in 64 (79%) of the 81 PCR-positive patients, while only 12 (7%) of the 164 PCR-negative patients demonstrated *H. pylori* positivity using this method. This was associated with taking biopsies from different localization of the antrum. In the PCR-positive group, most cases were of moderate severity (45/66, 68.2%), while in the PCR-negative group, 19 of the 24 cases of chronic gastritis (79.1%) were of mild severity. No cases of intestinal metaplasia or atrophy were observed. Among 81 cases, the amount of *H. pylori* staining was classified as +++ (severe) in 12 (14.8%), ++ (moderate) in 26 (32.1%), + (mild) in 26 (32.1%), and negative in 17 (21%) semi-quantatively. All *H. pylori* positive cases showed strong staining intensity. A comparison of the patients’ symptoms, endoscopic and histopathological findings according to the immunohistochemical test results is shown in Table 1.

**Table 1:** Comparison of symptoms, endoscopic and histopathological findings of the patients according to immunohistochemical test results

	Immunohistochemical test results				
	Negative (%)	+ (%)	++ (%)	+++ (%)	*p*
**Symptoms**					
Epigastric pain	13 (76.5)	19 (73.1)	17 (65.4)	10 (83.3)	
Vomiting	4 (23.5)	6 (23.1)	6 (23.1)	1 (8.3)	> 0.05
Dysphagia	0	1 (3.8)	3 (11.5)	1 (8.3)	
**Endoscopy**					
Gastric hyperemia	7 (41.2)	21 (80.8)	23 (88.5)	12 (100)	
LES relaxation	5 (29.4)	2 (7.7)	0	0	
Antral hyperemia	4 (23.5)	1 (3.8)	1 (3.8)	0	0.003
Antral nodularity	0	2 (7.7)	2 (7.7)	0	
Normal	1 (5.9)	0	0	0	
**Histopathology**					
Chronic gastritis	7 (41.2)	24 (92.3)	23 (88.5)	12 (100)	
Superficial gastritis	5 (29.4)	1 (3.8)	2 (7.7)	0	
Focal active gastritis	2 (11.8)	0	0	0	0.007
Lymphocytic gastritis	1 (5.9)	0	1 (3.8)	0	
Normal	2 (11.8)	1 (3.8)	0	0	

*LES* Lower esophageal sphincter

Bacteriological analysis revealed that 70 (28.57%) of the 245 biopsy samples were positive for *H. pylori* and a total of 71 *H. pylori* isolates were obtained from 70 samples (2 isolates from 1 sample).

The prevalence of the virulence genes of the *H. pylori* isolates was as follows: babA2 (78.9%), cagA (38%), cagE (54.9%), iceA1 (28.2%), iceA2 (19.7%), vacA m1 (25.4%), vacA m2 (70.4%), vacA m1m2 (1.4%), vacA s1 (56.3%), vacA s2 (38%), vacA s1a (64.8%), vacA s1b (8.5%), and vacA s1c (21.1%). The most prevalent gene was babA2 (78.9%), the least prevalent was vacA s1b (8.5%). In particular, the presence of the genes cagE, iceA2, vacA m1, vacA m2, vacA s1, vacA s2, vacA s1a, vacA s1b and vacA s1c differed between two different *H. pylori* isolates obtained from the same biopsy sample (sample no. 166). The simultaneous isolation of two different *H. pylori* strains from one biopsy sample was considered a mixed infection.

ERIC-PCR analysis revealed that all *H. pylori* isolates had band profiles consisting of 1 to 7 fragments between 150 bp and 1500 bp. All 71 *H. pylori* isolates in the study were successfully genotyped using the ERIC-PCR method and 30 different ERIC profiles were determined (Supplementary Fig. S1).

The *H. pylori* isolates were 77.46% (55/71) resistant to metronidazole, 26.76% (19/71) to clarithromycin, 14.08% (10/71) to levofloxacin and 5.63% (4/71) to rifampicin. On the other hand, all 71 isolates were sensitive to both amoxicillin and tetracycline. The detailed results, including the MIC values for each antibiotic, are shown in Table 2.

**Table 2:**
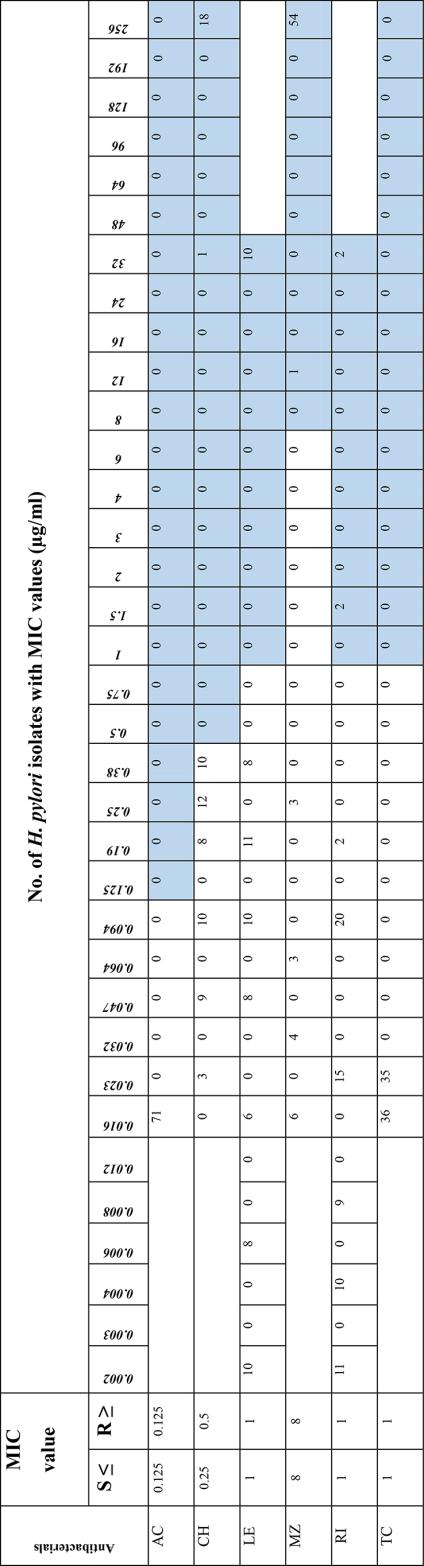
MIC distributions of *H. pylori* isolates (*n* = 71)

*AC* Amoxicillin (0.016-256); *CH* Clarithromycin (0.016-256); *LE* Levofloxacin (0.002-32); *MIC* Minimum inhibitory concentration; *MZ* Metronidazole (0.016-256); *R* Resistant; *RI* Rifampicin (0.002-32); *S* Sensitive; *TC* Tetracycline (0.016-256). Blue shades show the resistant isolates for each antibacterial tested

## Discussion

To our knowledge, this study is the first in our region to focus on this particular patient group and research topic. The most common symptoms were abdominal pain and vomiting. A meta-analysis revealed that epigastric pain was the most commonly reported symptom [14]. Gurbuz et al. found a higher prevalence of endoscopic biopsy in females and in the age group of 11–15 years [15]. Clinically, female adolescents were observed to suffer from abdominal pain more frequently than males, which could be related to their sensitivity to emotional stress [16].

In the current study, 70 (28.57%), 81 (33%) and 76 (31%) of the 245 biopsy specimens were positive for *H. pylori* by culture-based, molecular analysis and histopathological examination, respectively. In fact, the worldwide prevalence of *H. pylori* in children has been reported to be approximately 32.3% (with a range of 27.3% to 37.8%) [17].

Virulence factors such as vacA and cagA play an important role in the pathogenesis of *H. pylori*. In addition, genes such as babA2, cagE and iceA1 have also been reported to contribute to the pathogenesis of the disease. Nguyen et al. investigated the presence of cagA and vacA virulence genes in biopsy material from 268 pediatric patients and found a 69% prevalence of the cagA gene. The most common genotype was vacA s1/m2 (39.6%), the least common vacA s2/m1m2 (0.4%) [18]. In Türkiye, Saltik et al. found a prevalence of 55.6% for the cagA gene in *H. pylori* isolates from children and reported no significant correlation between cagA prevalence and the severity of gastro-duodenal lesions [19]. In the present study, the highest virulence gene detected was babA2 (78.9%), the lowest was vacA s1b (8.5%). The presence of the genes cagE, iceA2, vacA m1, vacA m2, vacA s1, vacA s2, vacA s1a, vacA s1b and vacA s1c also differed in the present study between two different *H. pylori* isolates from the same biopsy sample (sample no. 166). The differences in the prevalence of virulence genes could be due to variations in the number of samples analyzed and the type of materials used. For example, some studies rely on direct biopsy material, while others use isolated *H. pylori* strains, leading to different results [20].

PCR-based genotyping methods were used to genotype *H. pylori* isolates obtained from pediatric patients. Ozbey et al. classified 31 pediatric *H. pylori* isolates into four different genotypes using PCR-based restriction fragment length polymorphism (RFLP). They found no correlation between these genotypes and antral nodule formation and surmised that the limited number of PCR-RFLP profiles could be due to the small number of isolates analyzed [21]. In the present study, authors performed ERIC-PCR on 71 *H. pylori* isolates from 70 biopsy samples, resulting in the identification of 30 different ERIC profiles. This result indicates a high degree of genetic heterogeneity among the isolates. In the present study, the lower detection rate of *H. pylori* by immunohistochemical staining compared to PCR testing was interpreted as low bacterial density detected by PCR testing. The histopathological and endoscopic results showed a statistically significant increase in bacterial density, but the symptoms did not.

The extensive use of antibiotics worldwide has led to the development of resistance in numerous microbial groups, including *H. pylori*. Studies to determine the antibacterial resistance of *H. pylori* isolated from pediatric patients are limited both in Turkey and worldwide. The studies to detect antibiotic resistance in *H. pylori* isolates often use conventional phenotypic methods, which are considered the gold standard, and molecular methods that reveal the genetic basis of antibiotic resistance [22]. Ozçay et al. reported that 36.4% (12/33) of *H. pylori* isolates obtained by culture were resistant to metronidazole and 18.2% (6/33) were resistant to clarithromycin. All isolates tested were sensitive to tetracycline and amoxicillin [23]. Güven et al. observed that 27% of isolates were resistant to clarithromycin and 15% to fluoroquinolones [24], while Bahadori et al. reported a higher clarithromycin resistance rate of 56.75% using molecular methods [25]. In the review of Kaplan et al. metronidazole resistance was reported to be 50–76% [26]. In this study, 77.46% (55/71) of *H. pylori* isolates were resistant to metronidazole, 26.76% (19/71) to clarithromycin, 14.08% (10/71) to levofloxacin and 5.63% (4/71) to rifampicin. All isolates were sensitive to amoxicillin and tetracycline. The differences in resistance rates may be due to the number of isolates analyzed and the methods used to detect antibiotic resistance. It is also noteworthy that no resistance to amoxicillin and tetracycline was detected in index isolates, which is a promising result. However, due to the high rate of metronidazole resistance, it is crucial that metronidazole-free regimens are used in the empirical therapy of *H. pylori* in authors’ region.

In conclusion, this study is the first in authors’ region, considering the patient group and subjects. The data obtained are of epidemiological importance. In addition, the results of the antibiotic susceptibility test may be helpful in the selection of antibiotics for the treatment of *H. pylori* disease.

## Electronic Supplementary Material

Below is the link to the electronic supplementary material.

Supplementary Material 1
