# Supplementary figures and images for: Helicobacter pylori in Turkish Children with Dyspepsia: Diagnosis, Prevalence, Genotyping and Antibiotic Resistance
Source: Indian J Pediatr. 2025 Jul 5;93(1):17–22. doi: 10.1007/s12098-025-05635-2 (PMC12764497; doi:10.1007/s12098-025-05635-2)

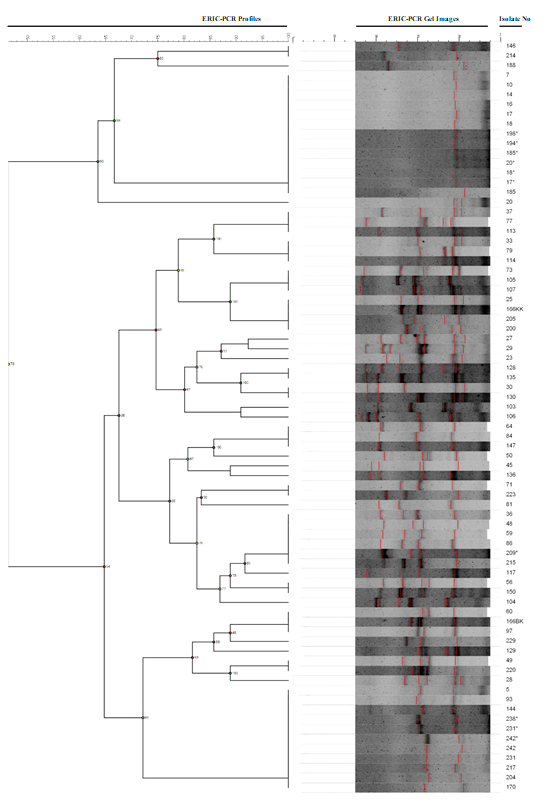


**Supplementary Fig. S1** The dendrogram constructed with ERIC-PCR results of 71 isolates

Supplement: Supplementary file 1 — Supplementary Material 1 [file 12098_2025_5635_MOESM1_ESM.docx]
